# A novel mechanism for A-to-I RNA-edited AZIN1 in promoting tumor angiogenesis in colorectal cancer

**DOI:** 10.1038/s41419-022-04734-8

**Published:** 2022-04-02

**Authors:** Yan Wei, Haowan Zhang, Qiaohui Feng, Shumin Wang, Youcheng Shao, Jie Wu, Ge Jin, Weiwei Lin, Xinxin Peng, Xiaoyan Xu

**Affiliations:** 1 Department of Pathophysiology, College of Basic Medical Science, China Medical University, Shenyang, 110122 Liaoning PR China; 2 Innovation Institute, China Medical University, Shenyang, 110122 Liaoning PR China; 3 Precision Scientific (Beijing) Co., Ltd., 100085 Beijing, PR China

**Keywords:** Epigenetics, Mechanisms of disease, Cancer microenvironment

## Abstract

Adenosine (A) to inosine (I) RNA editing catalyzed by adenosine deaminases acting on RNA (ADAR) enzymes is a post-transcriptional modification that emerged as a key player in tumorigenesis and cancer progression. Antizyme inhibitor 1 (AZIN1) is one of the most frequent A-to-I RNA alterations in many human cancers. RNA-edited AZIN1 is known to confer a gain-of-function phenotype associated with aggressive tumors. However, the functional impact of RNA-edited AZIN1 in cancer angiogenesis remains unexplored. We showed here that RNA-edited AZIN1 promoted tumor angiogenesis through the upregulation of IL-8 via in vitro and in vivo experiments. And we subsequently demonstrated that delaying c-Myc degradation by OAZ2-mediated ubiquitin-independent proteasome pathway contributed to increase mRNA level and the secretion of angiogenic factor IL-8. Our study suggests an important contribution of RNA-edited AZIN1 to the tumor vascular microenvironment and highlights its translational potential. Thus, we revealed a potential approach to explore small-molecule antagonists such as reparixin attenuating IL-8 signaling for treatment of human cancer patients detected with hyper-editing.

## Introduction

RNA editing is a widespread post-transcriptional nucleotide modification mechanism in humans and is catalyzed by adenosine deaminase acting on RNA (ADAR) enzymes. If RNA editing introduces specific nucleotide changes in the coding regions of mRNAs, even without altering the DNA sequence, it can lead to consequent amino acid substitutions in the protein. Not only transcriptome but also proteome diversities are expanded through RNA editing [1]. The most prevalent form of RNA editing involves the conversion of adenosine to inosine (A-to-I) by hydrolytic deamination at the C6 position of adenine, which is a widespread post-transcriptional mechanism in humans. Several RNA editing events show clinically relevant patterns; and importantly, the editing events in AZIN1 [2], COG3 [3], and COPA [1] can functionally drive the growth and migration of cancer cells in a manner similar to driver somatic mutations. A-to-I RNA editing contributes to protein heterogeneity at least in some cancer types, and thus deserves a greater degree of effort to clarify the molecular mechanism of human cancers and explore prognostic and therapeutic strategies.

Several recent studies show that antizyme inhibitor 1 (AZIN1) is one of the most common A-to-I RNA alterations in various cancer types, including hepatocellular carcinoma [2], non-small cell lung cancer [4], colorectal cancer (CRC) [5]. Mechanistically, edited AZIN1 conferred a gain-of-function phenotype associated with aggressive tumors [2, 5] and promoted ornithine decarboxylase (ODC) and polyamine accumulation [2]. Recent reports show that A-to-I editing of AZIN1 is intimately associated with augmented tumor-initiating potential and tumor-aggressive behavior. Tumor angiogenesis is known to play a critical role in tumorigenesis and tumor progression [6]. Angiogenesis, the formation of new blood vessels from the existing vasculature, is a hallmark of cancer that facilitates rapid tumor growth and metastasis [7]. Tumor angiogenesis is caused by an imbalance between pro- and anti-angiogenic cytokines. While most research on RNA-edited AZIN1 has centered on its role in the tumor rather than the tumor microenvironment, the biological function of RNA-edited AZIN1 on tumor angiogenesis has not been elucidated.

Here, through in vitro and in vivo experiments, we elucidate the molecular mechanism of RNA-edited AZIN1 in CRC angiogenesis through the regulation of IL-8 (or CXCL8). These findings define a novel function for RNA-edited AZIN1 in regulating tumor-induced angiogenesis. The need for fundamentally innovative approaches to the anti-angiogenic drugs was critically considered, not only by targeting well known growth factors like VEGF but also by targeting the newly validated factors of tumor-induced angiogenesis, which is vital for endothelial cells to form new vessels. Our work on the function of RNA-edited AZIN1 in tumor angiogenesis can provide further insights into how A-to-I RNA-edited AZIN1 promotes CRC development and progression and rationales for developing new therapies against tumor angiogenesis.

## Results

### Conditioned media from colorectal cancer cells overexpressing edited AZIN1 promotes HUVEC migration and tube formation

**Fig. 1:**
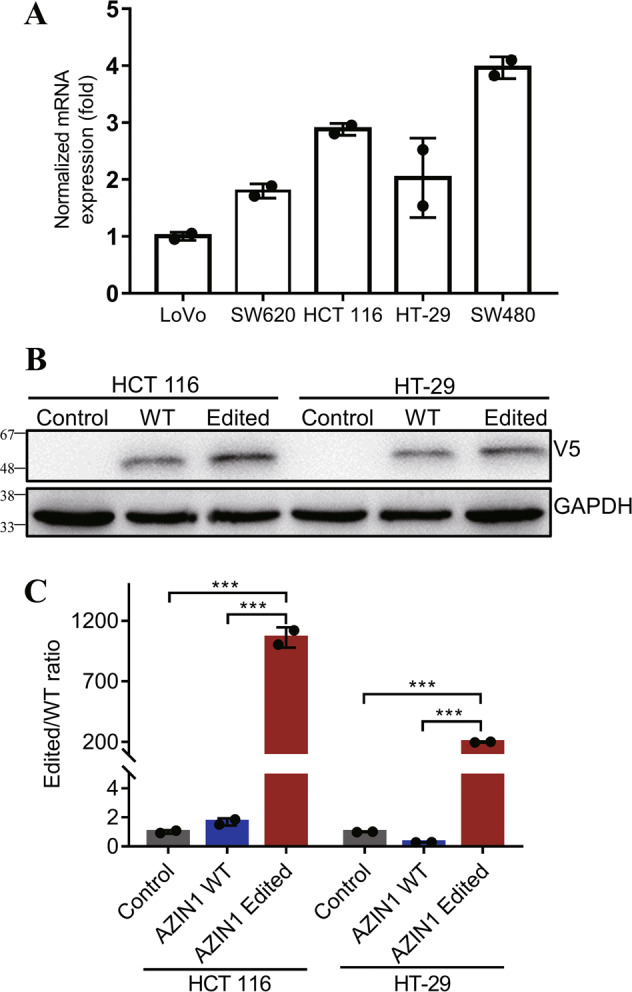
Validation of edited AZIN1 in colorectal cancer cell lines. **A** RT-qPCR of AZIN1 in different cell lines including HCT 116, HT-29, LoVo, SW480, and SW620. **B** Western blots of AZIN1 overexpression in HCT 116 and HT-29 cells. V5 tag was used to quantify the AZIN1 protein expression level, and GAPDH was used as loading control. (Control: control vector, WT: AZIN1 wild-type, Edited: AZIN1 edited-type). **C** Changes in AZIN1 editing level after transfection with different types of AZIN1 by RNA Editing Fingerprint Assay. Data are presented as means ± SD; *n* = 2; One-way ANOVA with Tukey’s test as post-hoc test was used to assess the difference; ***P < 0.001.

We confirmed AZIN1 mRNA expression in different CRC cell lines using RT-qPCR (Fig. 1A). To investigate the functional impact of edited AZIN1 in tumor-induced angiogenesis, we introduced V5-tagged WT AZIN1 and mutant AZIN1_S367G cDNAs into HCT 116 and HT-29 cell lines. The protein expression level of the exogenously introduced edited AZIN1 was similar to the WT AZIN1, as confirmed by western blot (Fig. 1B). The relative amount of edited/WT AZIN1 was assessed using RNA Editing Fingerprint Assay. Indeed, when mutant AZIN1 was overexpressed, the proportion of edited AZIN1 increased markedly (Fig. 1C).

**Fig. 2:**
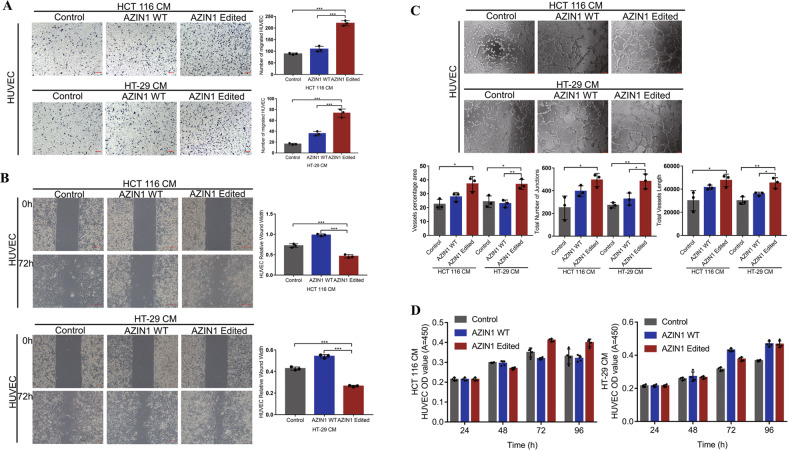
Functional effects of CM from colorectal cancer cells overexpressing edited AZIN1 on HUVEC. **A** Migration of HUVEC by treatment of CM from HCT 116 and HT-29 overexpressing edited AZIN1. Scale bars, 100 μm. **B** Wound healing of HUVEC. Scale bars, 200 μm. **C** Tube formation of HUVEC. Scale bars, 75 μm. **D** The proliferation of HUVEC tested by CCK-8. Data are presented as means ± SD; *n* = 3; One-way ANOVA with Tukey’s test as post-hoc test was used to assess the difference; *P < 0.05, **P < 0.01, ***P < 0.001.

We extracted the conditioned media (CM) from colorectal cancer cells for co-culture with HUVEC to explore whether enhanced RNA editing of AZIN1 from cancer cells can influence tumor angiogenesis. We found that CM from CRC cells overexpressing edited AZIN1_S367G significantly increased HUVEC migration, wound healing, and tube formation, in comparison with the control and WT AZIN1 groups (Fig. 2). First, we assessed cell migratory phenotypes of HUVEC cultured in CM from cancer cells transfected with the control vector, WT AZIN1, and edited AZIN1, by transwell assays and wound healing assays. A quantitative evaluation revealed a highly significant elevation in the number of cells migrating through the Boyden chamber when the medium in the lower chamber was CM from colorectal cancer cells transfected with edited AZIN1, whereas CM from WT AZIN1 showed no apparent difference, compared to the control group (Fig. 2A). The wound healing rate of HUVEC cultured in CM from the edited AZIN1 group was significantly higher than those cultured in CM from the control and WT AZIN1 cells (Fig. 2B). Moreover, HUVEC cultured in CM from the edited group also exhibited higher tube forming ability, as reflected in the increased percentage areas occupied by the vessels, the total number of junctions, and the total vessel lengths (Fig. 2C). These results verified that edited AZIN1 overexpression in cancer cells induced migration and tube formation of HUVEC in vitro.

### AZIN1 RNA editing increases angiogenesis in colorectal cancer in vivo

**Fig. 3:**
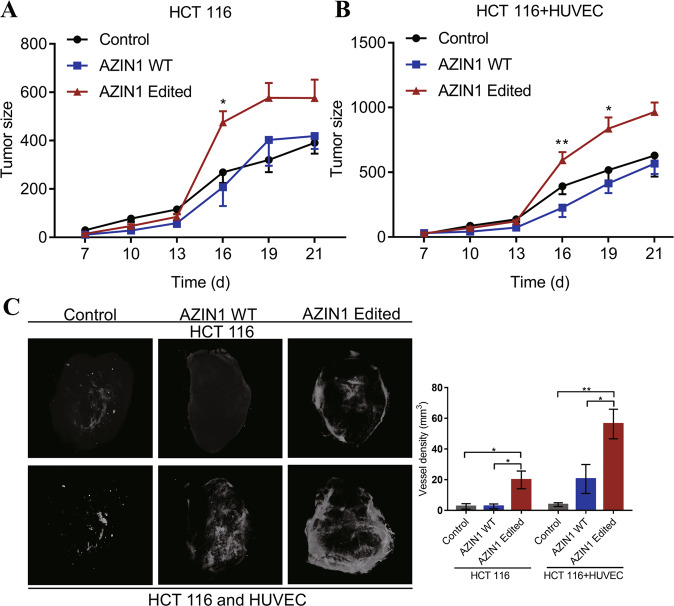
Effects of promoting tumor angiogenesis of edited AZIN1 in vivo. **A** HCT 116 cells were used in the xenograft model. **B** The mixture of HCT 116 and HUVEC cells were used in the xenograft model. Tumor sizes measured by caliper every 3 days in vivo model. **C** Representative images of intratumoral vessels by micro-CT. The vascular network was reconstructed using the CTAn software. Data are presented as means ± SEM. Data in **A** and **B** were analyzed with two-way ANOVA test with a Dunnett’s post hoc test. One-way ANOVA with Tukey’s test as post-hoc test was used to assess the difference of data in **C**. *P < 0.05, **P < 0.01, ***P < 0.001.

To assess the effect of edited AZIN1 in CRC progression, we performed xenograft experiments using AZIN1 overexpressing HCT 116 colon cancer cells mixed with HUVEC to confirm our in vitro findings. The results showed that with or without the HUVEC, volumes of the tumors derived from edited AZIN1-overexpressing HCT 116 groups were larger than those derived from the control and WT AZIN1 groups (Fig. 3A, B). Interestingly, tumor volumes were significantly higher in the group that received HCT 116 cells mixed with HUVEC rather than HCT 116 only group. Furthermore, and in accordance with our in vitro data, tumor vessel density was significantly increased in edited AZIN1-HCT 116 tumors compared to the control and WT AZIN1-HCT 116 groups, as determined by the micro-CT scans (Fig. 3C). The edited AZIN1 group showed evidence of a larger degree of vessel infiltration into the tumors, as reflected by their higher vessel density. These data support our in vitro findings and suggest that edited AZIN1 could prompt angiogenesis in the tumor.

### AZIN1 RNA editing prompts IL-8 secretion to promote angiogenesis

**Fig. 4:**
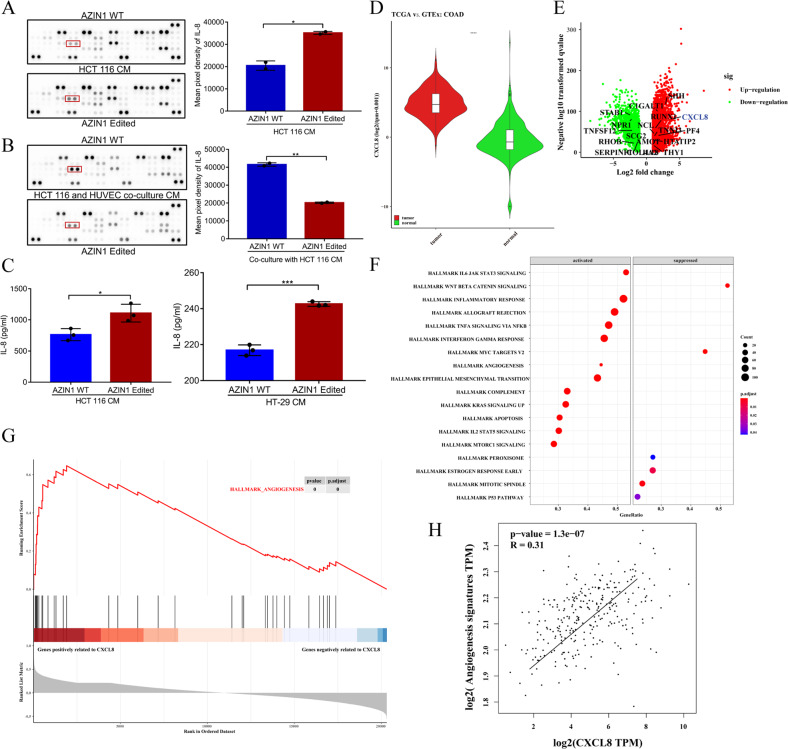
The differential factors secreted by edited AZIN1 compared with WT group. **A** Proteome profiler human angiogenesis array to detect the differential factors in HCT 116 cells and **B** co-culture of HCT 116 and HUVEC. Mean expression values of differential factors after densitometric quantification are shown. The grayscale value of IL-8 is circulated by a red rectangular box. **C** ELISA Quantitative determination of IL-8 from conditioned medium of each group. Data are presented as means ± SD. *n* = 3. Data were analyzed with unpaired Student’s *t* test. *P < 0.05, **P < 0.01, ***P < 0.001. **D** IL-8 mRNA expression was shown in colon cancer compared to para-cancer tissue. **E** Volcano plot revealed differentially expressed genes between colon cancer and para-cancer tissue. **F** Enrichment of genes with the hallmark pathways by GSEA analysis. **G** GSEA analysis was performed to identify the correlation of angiogenesis-related genes and IL-8. **H** Correlation between IL-8 and angiogenesis-related gene signatures. Spearman rank *p* value and correlation coefficient are shown.

Based on our results of increased edited AZIN1-induced angiogenesis, we extended the analysis to angiogenic cytokines released from cancer cells overexpressing edited AZIN1 using the Proteome Profiler Human Angiogenesis Array. A semi-quantitative analysis of the levels of 55 secreted angiogenesis-related cytokines in CM from the study groups revealed higher levels of IL-8 and VEGF-A in the edited AZIN1 group (Fig. 4A). Moreover, IL-8 was significantly decreased after co-culture with HUVEC in the edited AZIN1 group (Fig. 4B). Supporting this data, ELISA measurements revealed a significant upregulation of IL-8 in CM from cancer cells transfected with edited AZIN1 as compared to the WT AZIN1 group (Fig. 4C). The minor upregulation of VEGF-A in the edited AZIN1 CM was also verified by ELISA but did not reach significance (data not shown). These results suggest that edited AZIN1-induced angiogenesis may partly be due to increased secretion of the angiogenic factor IL-8 by these cells.

IL-8 (also called CXCL8) mRNA expression was higher in colon cancer compared to para-cancer tissue (Fig. 4D, E). To characterize angiogenesis-related function for IL-8 in colon cancer, GSEA analysis was conducted. As compared to the control group, angiogenesis pathways were significantly enriched in genes positively to IL-8 (Fig. 4F, G). To explore the relationship between IL-8 and angiogenesis process, correlation analysis was performed for subsequent study, and the analysis showed that angiogenesis-related gene signatures were significantly correlated with IL-8 (Fig. 4H).

**Fig. 5:**
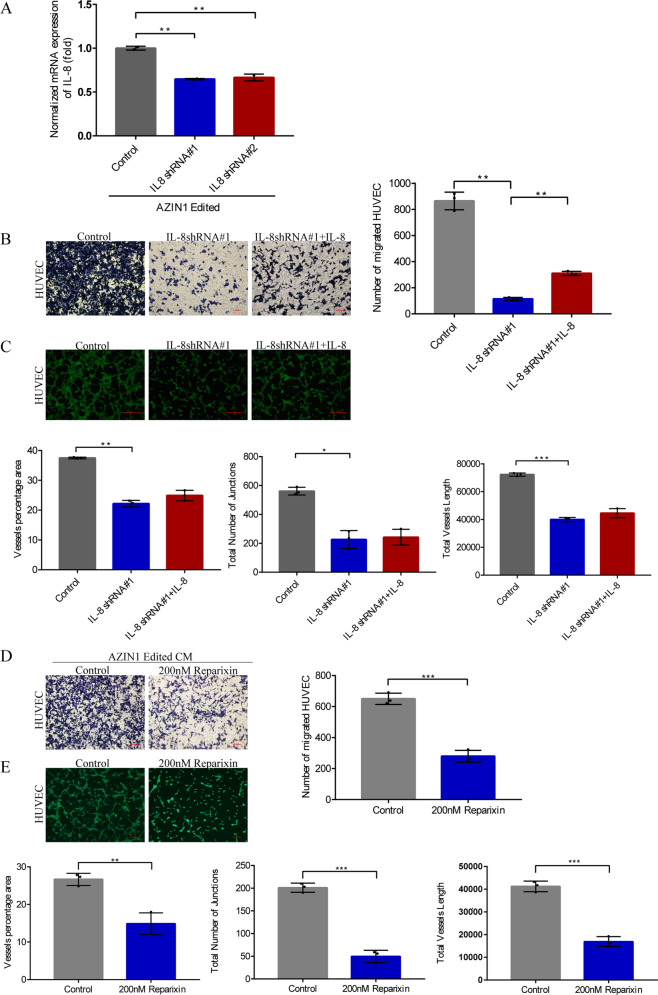
Knock-down of IL-8 decreased migration and tube formation of HUVEC. **A** RT-qPCR tested IL-8 mRNA level in IL-8 knock-down groups from HCT116 AZIN1 edited. **B** Migration of HUVEC. Scale bars, 100 μm. **C** Tube formation of HUVEC by treatment of CM from HCT 116 edited AZIN1 which IL-8 was knocked down by shRNA#1 or not. Scale bars, 500 μm. **D** Migration and **E** tube formation of HUVEC by treatment of reparixin. Scale bars, 100 μm. Data are presented as means ± SD. *n* = 3. One-way ANOVA with Tukey’s test as post-hoc test was used to assess the difference of data in **A**–**C**. Data in **D** and **E** were analyzed with unpaired Student’s *t* test. *P < 0.05, **P < 0.01, ***P < 0.001.

To further validate edited AZIN1 to induce angiogenesis through upregulation of IL-8, IL-8 shRNA#1 and IL-8 shRNA #2 were used to knockdown the expression of IL-8 in HCT 116 cells overexpressed edited AZIN1(Fig. 5A), and then the various groups of conditioned medium were acquired to cultivate HUVEC cells. We found that downregulation of IL-8 decreased migration and tube formation of HUVEC in comparison with the control group. Furthermore, adding cytokine IL-8 could slightly rescue tube formation and notably increase migration of HUVEC (Fig. 5B, C). Similarly, reparixin, which is an inhibitor of IL-8 receptor CXCR1 and CXCR2 activation, blocked the proangiogenic effects of IL-8 (Fig. 5D, E). These results support that edited AZIN1 induces angiogenesis through elevating secretion of IL-8.

**Fig. 6:**
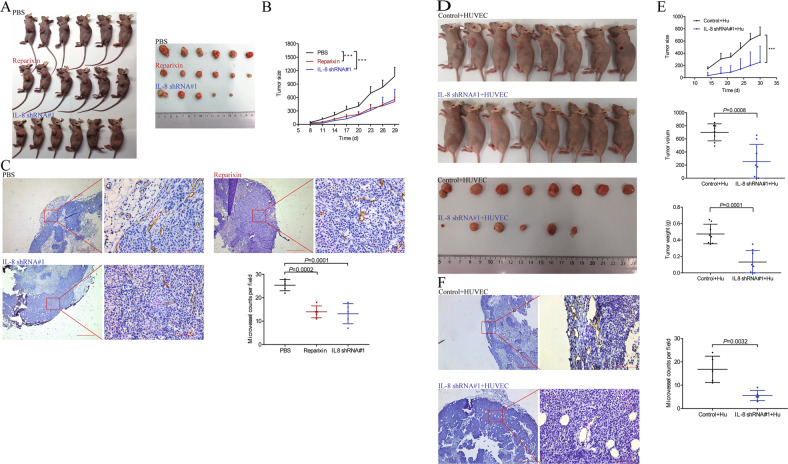
Effects of inhibiting tumor angiogenesis of IL-8 knock-down and reparixin in vivo. **A**, **D** The images of the mice and tumors. **B**, **E** Tumor sizes and weights were measured in vivo model. **C**, **F** The paraffin-embedded tumor tissue sections were immunostained with CD31. Representative tumors are shown. Original magnification, ×40 (scale bars: 200 μm) and ×400(scale bars: 20 μm); *n* = 5. Data in (**B**) are presented as means ± SEM. Data in (**B**) were analyzed with two-way ANOVA test with a Dunnett’s post hoc test. Data in (**C**–**F**) are presented as means ± SD. One-way ANOVA with Tukey’s test as post-hoc test was used to assess the difference of data in (**C**). Data in the first (**E**) were analyzed with two-way ANOVA test with a Dunnett’s post hoc test. Data in the other (**E**) and (**F**) were analyzed with unpaired Student’s *t* test. *P < 0.05, **P < 0.01, ***P < 0.001.

To assess the relationship between edited AZIN1 and IL-8 in CRC progression, we performed xenograft experiments using edited AZIN1 overexpressing HCT 116 cells transfected IL-8 shRNA#1 or negative scramble control to confirm our in vitro findings. The results showed that with or without the HUVEC, tumor sizes derived from IL-8 knock-down group were lower than those derived from the negative control group (Fig. 6A, B, D, E). To improve the likelihood and potential of clinical application, we also applied the inhibitor drug to treat mice suffering from the tumor by intraperitoneal (IP) injection of reparixin. The drug delayed the growth of tumors in mice compared with PBS injected group (Fig. 6A, B). Moreover, and in accordance with our in vitro data, when the harvested tumors were analyzed, xenograft tumors from the IL-8 knock-down group and reparixin treated group showed a significant decrease in CD31 staining by immunohistochemistry, compared to the control group (Fig. 6C, F).

### AZIN1 RNA editing increases IL-8 secretion through delaying c-Myc degradation

**Fig. 7:**
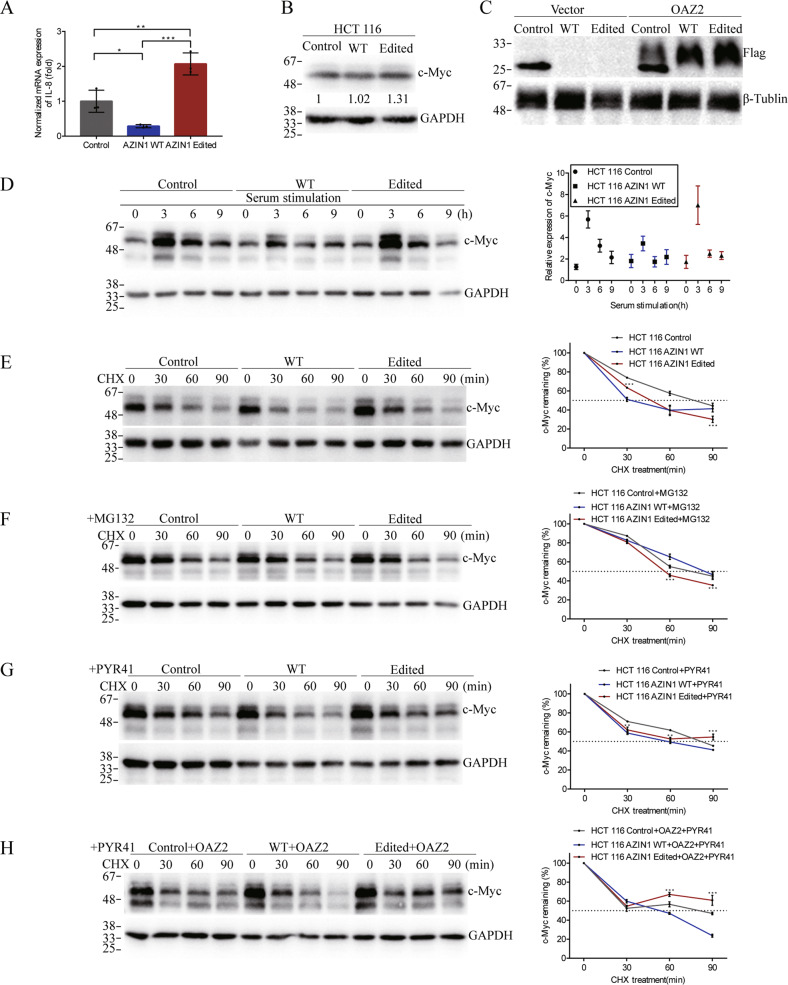
Analysis of c-Myc expression by Western blot (with β-tubulin and GAPDH as loading controls). **A** RT-qPCR of IL-8 and **B** Western blots of c-Myc in HCT 116 cells transfected with control, AZIN1 WT type, and AZIN1 edited type. **C** Western blots of OAZ2 overexpression in three groups of cells. Flag tag was used to quantify the expression of OAZ2 protein. **D** Western blots of c-Myc in cells stimulated by serum at indicated time point after starvation. **E** Western blots of c-Myc in cells treated with CHX. **F** Western blots of c-Myc in cells treated with CHX after pretreated with MG132 for 6 h. **G** Western blots of c-Myc in cells treated with CHX after pretreated with PYR41 for 6 h. **H** Western blots of c-Myc in OAZ2 overexpression cells treated with CHX after pretreated with PYR41 for 6 h. Data are presented as means ± SEM. Data in **A** and from **E**–**H** were analyzed with two-way ANOVA test with a Tukey’s multiple comparisons test. Only the significant results from edited group compared with WT group were marked with asterisks. **P < 0.01, ***P < 0.001.

The mRNA level of IL-8 in edited AZIN1 group was also higher than that in WT AZIN1 and control groups (Fig. 7A). Firstly, the mechanism of RNA-edited AZIN1 to upregulate mRNA level and protein expression of IL-8 was explored. C-Myc was known as the transcriptional factor of IL-8 was well identified in previous publication [8]. Compared with the WT AZIN1 group, the protein expression of c-Myc increased in edited AZIN1 overexpressed HCT 116 (Fig. 7B). In addition, edited AZIN1 notably promoted expression of c-Myc after serum stimulation (Fig. 7D). Then, we performed a cycloheximide chase experiment in the three groups of cell lines. Pharmacologic inhibition of de novo protein synthesis using cycloheximide (CHX, 50 μg/mL) in three groups of cells led to a time-dependent decrease in c-Myc protein expression. Intriguingly, c-Myc presented much longer half-lives in edited AZIN1 group than WT group (Fig. 7E). The above results showed that the edited AZIN1 delayed the degradation of c-Myc, thereby promoting the transcription of IL-8, which increased mRNA level and then protein expression of IL-8.

Next, the mechanism of editing AZIN1 delay c-Myc degradation was further detected. Three groups of cells were treated by MG132 (a proteasome inhibitor) for 6 h, and then CHX was added. After proteasome pathway was blocked by MG132, c-Myc protein could still degrade through non-proteasome pathway, although the rate of degradation slowed down in three groups because of the treatment of MG132 to inhibit proteasome degradation pathway, compared with the CHX treatment only, especially at the time point of 30 min by CHX treatment (Fig. 7F). It indicated that protein degradation pathway of c-Myc in this process at least includes the degradation pathway of proteasomes and non-proteasomes. Never the less, c-Myc regulation was not significantly different between edited AZIN1 and WT in the non-proteasome pathway around 30 min of CHX treatment (Fig. 7F). Compared with the WT group, there was no obvious superiority for the edited group in the performance of delaying c-Myc protein degradation in the regulation of non-proteasome pathways, where degradation is a little faster in edited group than that in WT group (Fig. 7F). Then, how edited AZIN1 delayed c-Myc degradation via the proteasome pathway was further investigated. Following the exposure of PYR41 (40 μM), an inhibitor of the E1 ubiquitin-activated enzyme [9], to block ubiquitinated proteasomal degradation pathway, the edited group notably delayed c-Myc degradation in the non-ubiquitinated proteasome degradation pathway (Fig. 7G). This suggested that edited AZIN1 delayed the degradation of c-Myc, at least went through the non-ubiquitin-dependent proteasome degradation pathway, compared to WT AZIN1. Moreover, based on the report that AZIN1 could bind to OAZ2 [10], and OAZ2 could mediate c-Myc non-ubiquitin-dependent proteasome degradation pathway [11], we introduced OAZ2 to excavate whether OAZ2 could play a role in this process. Here, after exogenous overexpression of OAZ2 (Fig. 7C) and treatment with PYR41, the degradation rate of c-Myc was obviously delayed, and even there was a little promotion of c-Myc expression in edited group, compared with WT group (Fig. 7H). It indicated that compared with WT AZIN1, RNA-edited AZIN1 may delay c-Myc degradation through OAZ2 in the ubiquitin-independent proteasome pathway (Fig. 7H). These results suggested that edited AZIN1 delayed degradation of c-Myc by OAZ2-mediated the ubiquitin-independent proteasome pathway to some extent, which promoted IL-8 transcription, and then upregulated mRNA level and protein expression of IL-8.

## Discussion

A single nucleotide change induced by editing in the coding region can markedly alter protein function. Here, we focused on an A-to-I editing event in the coding gene AZIN1, which is edited by ADAR1 [2, 12], and characterized its functional consequences in the vascular microenvironment of CRC. Edited AZIN1 is known to promote cancer cell proliferation and tumor progression through restraining antizyme-mediated degradation of oncoproteins, such as ODC and cyclin D1 [2, 4, 5, 13]. Edited AZIN1 exhibits a gain-of-function phenotype involving aggressive tumor behaviors. More recently, it has also been proposed that abnormal tumor vasculature may contribute to a hypoxic tumor microenvironment and hence maintain tumor cells in an invasive state [14]. Additionally, the vascular and lymphatic systems facilitate tumor spread [15]. Despite the growing evidence for A-to-I RNA editing in tumorigenesis, the functional role and the clinical significance of AZIN1 RNA editing in CRC tumor angiogenesis remains unexplored.

The significant effect of edited AZIN1 on tumor vascular microenvironment provides a notable rationale for further exploring how to target tumor vasculature to achieve better treatment efficacy. Here, we first explored the role of edited AZIN1 in the tumor vascular microenvironment of CRC. Overexpression of edited AZIN1 significantly promoted migration and tube formation in HUVEC; although, culturing HUVEC in CM from different groups did not affect their proliferation (Fig. 2D). Moreover, nude mice injected with cells expressing edited AZIN1 exhibited faster tumor growth and tumor angiogenesis. We also identified angiogenic chemokine IL-8 as possible angiogenesis mediator in the CM of edited AZIN1-containing cells.

The chemokine IL-8, with a defining CXC amino acid motif, was initially characterized for its leukocyte chemotactic activity [16]. IL-8, which binds to two G-protein coupled receptors, CXCR1, and CXCR2, is now known to also possess tumorigenic and proangiogenic properties [17], and our experimental results also offered the giant support for the proangiogenic role of IL-8. Reparixin, which is an inhibitor of IL-8 receptor CXCR1 and CXCR2 activation [18], was applied to inhibit the function of IL-8. The expression of IL-8 receptors on cancer cells, endothelial cells, neutrophils, and tumor-associated macrophages suggests that the secretion of IL-8 from cancer cells may have a profound effect on the tumor microenvironment. The activation of IL-8 receptors on endothelial cells promotes angiogenic response in endothelial cells and increases the proliferation, survival, and migration of vascular endothelial cells [19]. IL-8 can also disrupt cell junctions, leading to increased permeability of endothelial cells [20]. IL-8 signaling promotes the transactivation of epidermal growth factor receptor (EGFR) in vascular endothelial cells and activation of MAPK signaling [21]. IL-8 expression correlates with the angiogenesis, tumorigenicity, and metastasis of tumors in numerous xenograft models [22–24]. Moreover, IL-8 can enhance tumor growth by inducing a significant increase in CD31^+^ peritumoral vasculature in IL-8 transgenic mice [20, 25, 26]. Here, increase of IL-8 in the CM indicated that edited AZIN1 could enhance angiogenesis by affecting the release of IL-8. Furthermore, blood vessels have angiocrine capacity and can support the growth of tumors directly through the production of cytokines and growth factors [27]. As our study shows, IL-8 secretion changed after HUVEC co-culture with cancer cells. We also analyzed that IL-8 was correlated positively with markers of angiogenesis in CRC from data in TCGA. In addition, both IL-8 shRNA transfected in edited AZIN1 group and drug reparixin inhibited migration and tube formation of endothelial cells, and IL-8 addition could rescue the depressed performance to some extent. The experimental results in vivo also supported the experimental conclusions in vitro. All these previous findings are well aligned with the mechanistic model we propose in which the functional impact of edited AZIN1 is at least partially due to increased expression of IL-8, which promotes migration and tube formation of endothelial cells. In addition to drugs that target angiogenesis-related cytokines, thus the role of inhibitors related to vascular cytokine receptor like reparixin is also gaining much attention on tumor blood vessels.

Three antizyme isoforms are conserved among mammals. Ornithine Decarboxylase Antizyme 1 (OAZ1) and Ornithine Decarboxylase Antizyme 2 (OAZ2) have a broad tissue distribution, while OAZ3 is testis specific [28]. Both OAZ1 and OAZ2 inhibit cellular polyamine uptake and bind to AZIN1, which is homologous to ODC but lack its catalytic activity [29, 30]. Moreover, OAZ1 and OAZ2 accelerate ODC degradation in living cells, but OAZ2 binds to ODC less tightly than does OAZ1 [10]. In the present study, we show for the first time that RNA-edited AZIN1 delayed degradation of c-Myc by OAZ2-mediated the ubiquitin-independent proteasome pathway, which promoted IL-8 transcription, and then upregulated mRNA level and protein expression of IL-8. Prior work has shown that RNA-edited AZIN1 promoted cancer cell proliferation and tumor progression through restraining antizyme-mediated degradation of oncoproteins, such as ODC and cyclin D1 [2]. AZIN1 could bind to OAZ2 [31], and OAZ2 rather than OAZ1 could directly bind to c-Myc, and accelerate c-Myc degradation in an ubiquitin-independent manner and affect the pre-rRNA synthesis by regulating c-Myc level [11]. C-Myc, as one of transcriptional factors of IL-8, could increase the transcriptional level of IL-8 [8]. Our results that compared with WT group, the editing group notably delayed c-Myc degradation by the non-ubiquitinated proteasome degradation pathway in the presence of the E1 inhibitor PYR41, and obviously delayed the degradation rate of c-Myc, and even elevated of c-Myc expression after exogenous overexpression of OAZ2 and exposure of PYR41, provide strong evidence that RNA-edited AZIN1 delayed degradation of c-Myc by OAZ2-mediated the ubiquitin-independent proteasome pathway.

**Fig. 8:**
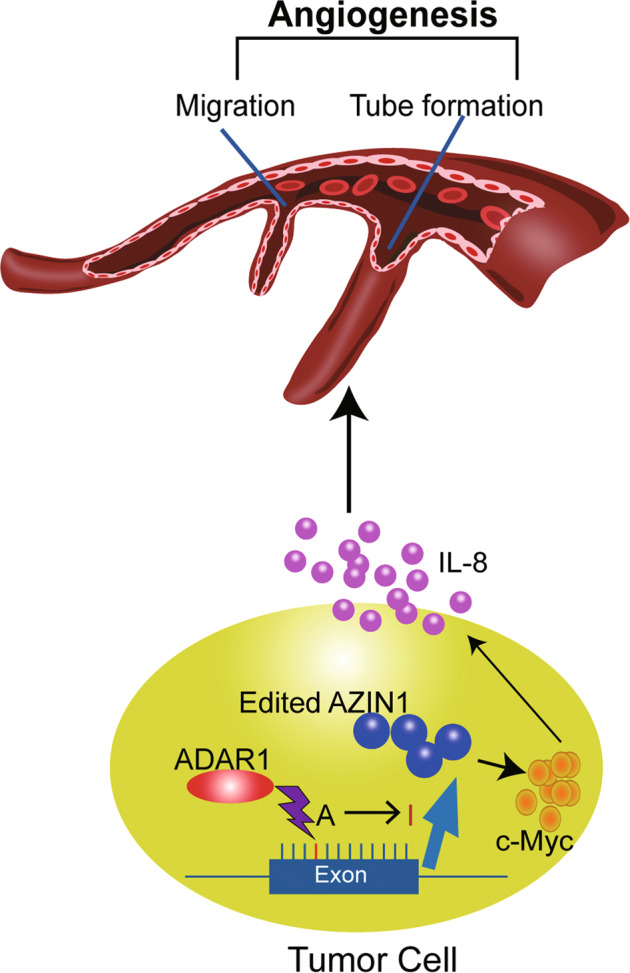
Schematic summary of the proposed mechanism of edited AZIN1 in promoting tumor angiogenesis. RNA-edited AZIN1 promoted tumor angiogenesis through the upregulation of IL-8, and delaying c-Myc degradation contributed to increasing IL-8 secretion.

Finally, A-to-I RNA editing of AZIN1 is critical for migration and tube formation of endothelial cells, which is indispensable to the formation of new vessels in development and disease, especially in tumorigenesis and progression. A-to-I RNA-edited AZIN1 promotes angiogenesis mainly by upregulation of the angiogenic factor IL-8 (Fig. 8). Regulation of c-Myc, a known transcriptional factor of IL-8, by RNA-edited AZIN1, is novel connection between the RNA-edited AZIN1 and tumor angiogenesis. C-Myc is constitutively and aberrantly expressed in over 70% of human cancers. The direct inhibition of c-Myc has been shown to trigger rapid tumor regression in mice, which may be a viable therapeutic approach [32], and moreover, targeting IL-8 may also be a precise and reasonable strategy to inhibit tumor angiogenesis. This renders IL-8 an attractive target for suppressive therapy of tumor angiogenesis. Accordingly, inhibiting the pronounced effects of IL-8 signaling within the tumor vascular microenvironment may have significant therapeutic potential in modulating tumor progression. Furthermore, based on our work, it would be interesting in the future to perform extensive preclinical investigations by applying small molecule antagonists such as reparixin and humanized monoclonal antibodies attenuating IL-8 signaling for treatment of human cancer patients detected with hyper-editing.

## Materials and methods

### Cell culture and conditioned media

HCT 116, HT-29, LoVo, SW480, SW620, and HEK 293 cells were obtained from National collection of authenticated cell cultures and Cell Bank, Chinese Academy of Sciences (Shanghai, China). HUVEC cells were obtained from Procell Life Science & Technology Co., Ltd. All cell lines were confirmed by short tandem repeat analysis and were mycoplasma negative. All colorectal cancer cells were maintained in RPMI 1640 (Hyclone, USA) supplemented with 10% fetal bovine serum (FBS) (CLARK, Uruguay). HEK 293 and HUVEC were cultured in high glucose DMEM (Hyclone, USA) supplemented with 10% FBS. All cells were cultured in a humidified atmosphere at 37 °C and 5% CO_2_. All cell-based experiments were conducted within 6 passages after thawing. To generate conditioned media (CM) from CRC cells, HCT 116 or HT-29 cells were cultured in complete growth medium first. When the cells reached 90% confluence, the medium was replaced with serum-free DMEM. The supernatant was collected as CM after 72 h. To detect whether edited AZIN1 prevented c-Myc to degrade, cells were treated with 50 μg/ml cycloheximide (CHX, GC-17198 from GLPBIO) for 30 min, 60 min and 90 min. Cells were also stimulated at 3 h, 6 h and 9 h by serum after starvation for 24 h. To detect the how edited AZIN1 delayed c-Myc to degrade, cells were treated with 20 µM MG-132 (HY-13259 from MCE) or 40 µM PYR-41 (GC-15771 from GLPBIO) for 6 h before CHX.

### Lentiviral package and cell transfection

The mutant AZIN1 (S367G) open reading frame corresponding to the RNA editing site in AZIN1 was generated using site-directed mutagenesis and confirmed by Sanger sequencing, as previously described [3]. Viruses were produced by transfection of HEK293 cells with the pHAGE-puromycin control vectors, pHAGE-V5-puromycin expression vectors (carrying AZIN1-WT or AZIN1_S367G), pLVX-shRNA1 negative scramble control vectors, pLVX-IL-8 shRNA#1 (carrying sequence: CCGGCAAGGAGTGCTAAAGAACTTACTCGAGTAAGTTCTTTAGCACTCCTTGTTTTTG) or pLVX-IL-8 shRNA#2 (carrying sequence: CCGGCCGAACTTTAATTTCAGGAATCTCGAGATTCCTGAAATTAAAGTTCGGTTTTTG), and the lentiviral packaging plasmids psPAX2 and pMD2G. HCT 116 and HT-29 cells were transduced by the virus followed by selection with puromycin (HCT 116, 1 μg/mL; HT-29, 2 μg/mL), and after 7 days of antibiotic selection, expression of the constructs was verified by RT-qPCR, western blots and RNA editing fingerprint assay.

Cells were transfected with pCMV6-Entry control vector and pCMV6 expression vector carrying OAZ2 (NM_002537) human tagged ORF, using jetOPTIMUS^®^ transfection reagent (101000006 from Polyplus), and expression of the constructs was verified by Western blots.

### Western blot

Protein lysates were extracted in RIPA buffer (25 mM pH 7.6 Tris-HCl, 150 mM NaCl, 1%NP-40, 1% sodium deoxycholate, 0.1% SDS) containing protease and phosphatase inhibitors (Beyotime, P1050). Protein lysates were quantified, separated by SDS-PAGE gel, transferred onto polyvinylidene difluoride (PVDF) membranes (Millipore, Billerica, MA, USA), and detected with specific primary antibodies and HRP-conjugated secondary antibodies in combination with enhanced chemiluminescence (Vazyme, E411-04). ImageJ was used for densitometric quantification of the acquired signal, and results were normalized to control. The following antibodies were used: V5 (1:5000; R960-25, Life Technologies), DYKDDDDK tag polyclonal antibody binding to FLAG tag epitope (1:5000; 20543-1-AP, proteintech), c-Myc (1:1000; E5Q6W, Cell Signaling Technology) and β-tubulin (1:3000; 100109-MM05T, Sino Biological) and GAPDH (1:3000; sc-25778, Santa Cruz Biotechnology).

### RT-qPCR and RNA editing fingerprint assay

Lentivirus-transduced cells were harvested, and total RNA was isolated using MiniBEST Universal RNA Extraction Kit (TaKaRa, 9767) according to the manufacturer’s instructions. RNAs were transcribed into cDNAs using the PrimeScript RT reagent Kit with gDNA eraser (TaKaRa, RR047A). We designed RNA editing site-specific primers that were compatible with SYBR green RT-qPCR protocols, according to a previous study describing the RNA editing fingerprint assay [33]. We performed qPCR in triplicate using the transcribed cDNA on an Applied Biosystems StepOne^TM^ Real-Time PCR system (Applied Biosystems, Darmstadt, Germany) and TB Premix Ex Taq II (TaKaRa, RR820A). Melting curve analysis was performed on each plate according to the manufacturer’s instructions. The relative expression was calculated by the 2^(–DDCt)^ method, and the expression levels were normalized to β-actin. The relative RNA editing level (edit/WT RNA ratio) was calculated as 2^(CtEdit – CtWT)^. The primers 5’-3’ from Nanjing springen Biotechnology were as follows: AZIN1 primers(forward: ACTGAATGACATCATGTAATAAATGGCT; reverse: AATACAAGGAAGATGAGCCTCTGTTTAC), AZIN1 WT type primers(forward:CATTCAGCTCAGGAAGAAGACATCT; reverse: AATACAAGGAAGATGAGCCTCTGTTTAC), AZIN1 edited type primers(forward: ACTGAATGACATCATGTAATAAATGGCT; reverse: GAGCTTGATCAAATTGTGGCAG), IL8 primers(forward:GAGAGTGATTGAGAGTGGACCAC; reverse:CACAACCCTCTGCACCCAGTTT) and β-actin primers(forward: ATTGGCAATGAGCGGTTCCG; reverse: CGTGGATGCCACAGGACTCC).

### Cell proliferation assay

Cell counting kit-8 (CCK8, MCE) was used to assess the proliferation of HUVEC cultured in CM from CRC cells. HUVEC (1 × 10^3^/well) were seeded into 96-well plates. After 24 h, the growth medium was replaced with 100 μl CM. After 24, 48, 72, 96 h of seeding, a batch of cells was stained with 11 μl of CCK8 reagent at 37 °C for 2 h. The optical density (OD) was quantified with an automatic plate reader (Multiskan Sky, Thermofisher, USA) at 450 nm. Experiments were performed in quadruplicate for each condition tested.

### Scratch assay in HUVEC cultured with CM from CRC cells

HUVEC (2 × 10^5^) were seeded on the bottom of a 12-well plate. After 24 h, when cells were 90% confluent, the monolayers were scratched using a sterile 10 μl micropipette tip to create a wound ~300 μm across. After washing, CM from CRC cells was added. Images of the wound in each well were acquired at 0, 24, 48, and 72 h under a phase-contrast microscope (Leica, Germany) at 200× magnification. Wound edges were used to calculate wound area at 0 and 72 h. Results are expressed as a percentage of the initial wound area (0 h).

### Migration assay in HUVEC cultured with CM from CRC cells

Cell migration was assessed by Transwell assay. Briefly, 1 × 10^5^ HUVEC suspended in serum-free DMEM were seeded in the upper chamber (8 μm pores, Corning, USA) of the 24-well transwell chamber. CRC cell CM was used as a chemoattractant in the lower chamber. After 24 h of incubation, migrated cells were fixed with methyl alcohol and stained with Coomassie brilliant blue. The stained cells were photographed under a microscope (Nikon, Japan) at 200× magnification. The number of migrated HUVEC was counted using Image-Pro Plus.

### Matrigel HUVEC tube formation assay

HUVEC (3 ×10^4^/well) were suspended in CM or CM added 10 ng/ml IL-8 (novoprotein, China) or 200 nM reparixin (GLPBIO, USA)and seeded onto Matrigel-coated wells of a 96-well plate precoated (Corning, USA). After 24 h of incubation, HUVEC cells were directly imaged without staining or stained by 5 nM eBioscience™ Calcein AM Viability Dye (Invitrogen, USA) for 30 min, and detected at 495 nm by fluorescent microscopy. And tube formation was assessed by estimating vessel percentage area, the total number of junctions, and total vessel length by AngioTool [34].

### Proteome profiler human angiogenesis array

We used the Proteome Profiler Human Angiogenesis Array (ARY007, R&D Systems, USA) for a qualitative comparison of 55 angiogenic cytokines among the different CM. The culture supernatants from each sample were incubated with the reconstituted detection antibody cocktail. The 55 different angiogenesis antibodies were spotted in duplicate onto the array membrane and incubated with the detection antibody mixture overnight at 4 °C. The membrane was then incubated in streptavidin-horseradish peroxidase conjugate, followed by a chemiluminescent detection reagent. The membrane was scanned using a chemiluminescence detection apparatus, and profiles of mean spot pixel density were created. The level of expression of each cytokine is directly proportional to the luminescence intensity. Data are reported as the mean pixel density of each cytokine. The experiment was performed twice in duplicate.

### Enzyme-linked immunosorbent assay (ELISA)

For quantification of the two differentially expressed cytokines, IL-8 (Elabscience, E-EL-H6008) and VEGF-A (Elabscience, E-TSEL-H0026) ELISA kits were used according to the manufacturer’s instructions. Standard or CM was added to each coated well and was incubated for 2 h at 37 °C. Then, plates were incubated with the biotinylated antibody for 1 h at 37 °C and then with HRP-avidin for 1 h at 37 °C. After additional washing steps, TMB substrate was added to each well for 15 min at 37 °C, following which, a stop solution was added for color development. The plates were read at 450 nm with an automatic plate reader (Multiskan Sky, Thermofisher, USA). Standard curves were generated using kit-provided standards and were used to calculate cytokine concentrations in the CM.

### Tumor xenograft

Immunodeficient mice (six-week-old female BALB/c-nu) were purchased from Liaoning Changsheng biotechnology co., Ltd and SPF (Beijing) Biotechnology Co., Ltd. To assess the involvement of AZIN1 RNA editing in tumor growth and angiogenesis, immunodeficient mice were injected subcutaneously in the right flank with 3 ×10^6^ HCT 116 cells and in the left flank with 3 × 10^6^ HCT 116 cells mixed with 6×10^5^ HUVEC (*n* = 8 mice per group). Next, RNA-edited AZIN1 prompted tumor growth and angiogenesis through increase of IL-8 was also identified by xenograft mouse model. At first, 3 × 10^6^ overexpressed edited AZIN1 HCT 116 cells or overexpressed edited AZIN1 HCT 116 cells transfected IL-8 shRNA#1 immunodeficient mice were injected subcutaneously in the flank of mice. Reparixin, a dose of 30 mg/kg/mouse was delivered intraperitoneally at once a day after 8 days of cell injection, and PBS solution was injected as the control (*n* = 6 mice per group). We also injected subcutaneously in the left flank of mice with 2 × 10^6^ overexpressed edited AZIN1 HCT 116 cells transfected negative scramble vectors or overexpressed edited AZIN1 HCT 116 cells transfected IL-8 shRNA#1 mixed with 4 × 10^5^ HUVEC (*n* = 8 mice per group). Tumor volume (mm^3^) was measured twice a week using the formula: tumor volume/size = 0.5 × length × width^2^. All animal experiments were performed in accordance with protocols approved by the Animal Ethics Committee of China Medical University (Shenyang, Liaoning, China). The person who provided animal care and measured tumor growth was blinded to the group allocation during all animal experiments and outcome assessment.

### Micro-CT scanning

Micro-CT **(**Skyscan1276, Bruker) was used for contrast imaging to assess the distribution of tumor blood vessels and compare the vessel densities between groups. Mice were sacrificed when they reached preset criteria (cachexia and emaciation). Each mouse was injected intraperitoneally with 1% sodium pentobarbital (50 mg/kg), and then 0.5 ml iopamidol was injected via inferior vena cava. After iopamidol had circulated for 5 min, the mouse was treated by euthanasia. The xenograft tumor was separated and placed in Micro-CT for scanning. CTAn software was used to reconstruct the vascular network of the tumor in three dimensions and calculate the vascular density.

### Immunohistochemistry

Paraffin-embedded tissue blocks were sectioned into 4μm-thick sections for IHC staining. Sections were then deparaffinized and rehydrated. For antigen retrieval, the slides were immersed in EDTA buffer (PH 9.0) and boiled for 10 min in an autoclave twice. Nonspecific binding was blocked with TBS containing 10% normal FBS and 1% BSA for 2 h at 37 °C. The slides were then incubated in 15 μg/ml CD31 antibody (R&D Systems, AF3628) at 4 °C overnight in a humidified chamber. Endogenous peroxidase activity was blocked with 0.3% hydrogen peroxide (H_2_O_2_) for 30 min. The slides were then incubated with HRP-conjugated donkey anti-goat antibody (1:2000, Jackson ImmunoResearch, 705-035-003) for 1 h at room temperature. The 3,5-diaminobenzidine (DAB) Kit (MXB, DAB-0031) was used for color development, and Mayer’s hematoxylin was used as a counterstain. Stained slides were visualized using a microscope (Nikon 90i) with 40× and 400× magnification.

### Bioinformatics analysis of IL-8 in COAD

We downloaded colon cancer and para-cancer sample datasets in the present study with complete expression profiles, and curated clinical information from PanCanAtlas (https://gdc.cancer.gov/about-data/publications/pancanatlas). Genes with zero expression were excluded in all samples and all gene names were remapped to official gene symbols according to the Multi-symbol checker tools (https://www.genenames.org/tools/multi-symbol-checker/). Differential expression was calculated using the R package DESeq2 using default parameters. Adjusted *P* < 0.05 and the fold change (FC) was ≥ 2-fold higher or lower (log FC > 1) were set as the cutoff criteria. R package UCSCXenaShiny [35] was used to perform the expression of IL-8 mRNA in pan-cancer and colon cancer, respectively. Gene set enrichment analysis (GSEA), which showed a significant improvement in the use of gene expression profile data, was performed by R package clusterProfiler [36] with default parameters using MSigDB H: hallmark gene sets (50 gene sets available, V7.4). Gene sets with a false discovery rate (FDR) value <0.05 was considered as significantly enriched. The angiogenesis gene signature was downloaded from the MSigDB database (http://www.gsea-msigdb.org/gsea/msigdb/index.jsp, GO:0001525). Subsequently, the Gene Expression Profiling Interactive Analysis (GEPIA) web server [37] was conducted to perform correlation analysis between IL-8 and angiogenesis gene signature.

### Statistical analysis

Prism software (GraphPad) was used for all statistical analyses. Differences between groups in vitro experiment results were evaluated using Student’s *t* tests when only 2 groups were analyzed or by one-way analysis of variance (ANOVA) when more than 2 groups were compared. In vivo tumor size comparison across different time points were compared with two-way ANOVA, each measurement was considered independent, and the treatment *P* value was reported. Data were presented as means ± SD, if not otherwise specified. *P* values lower than 0.05 was considered statistically significant.

## Supplementary information

Original western blots

Reproducibility Checklist

## Data Availability

The datasets used and/or analyzed during the current study are available from the corresponding author on reasonable request.

## References

[1] Peng X, Xu X, Wang Y, Hawke DH, Yu S, Han L (2018). A-to-I RNA editing contributes to proteomic diversity in cancer. Cancer Cell.

[2] Chen L, Li Y, Lin CH, Chan TH, Chow RK, Song Y (2013). Recoding RNA editing of AZIN1 predisposes to hepatocellular carcinoma. Nat Med.

[3] Han L, Diao L, Yu S, Xu X, Li J, Zhang R (2015). The genomic landscape and clinical relevance of A-to-I RNA editing in human cancers. Cancer Cell.

[4] Hu X, Chen J, Shi X, Feng F, Lau KW, Chen Y (2017). RNA editing of AZIN1 induces the malignant progression of non-small-cell lung cancers. Tumour Biol J Int Soc Oncodev Biol Med.

[5] Shigeyasu K, Okugawa Y, Toden S, Miyoshi J, Toiyama Y, Nagasaka T (2018). AZIN1 RNA editing confers cancer stemness and enhances oncogenic potential in colorectal cancer. JCI insight.

[6] Folkman J, Merler E, Abernathy C, Williams G (1971). Isolation of a tumor factor responsible for angiogenesis. J Exp Med.

[7] Hanahan D, Weinberg RA (2011). Hallmarks of cancer: the next generation. Cell..

[8] Florczyk U, Czauderna S, Stachurska A, Tertil M, Nowak W, Kozakowska M (2011). Opposite effects of HIF-1α and HIF-2α on the regulation of IL-8 expression in endothelial cells. Free Radic Biol Med.

[9] Yang Y, Kitagaki J, Dai RM, Tsai YC, Lorick KL, Ludwig RL (2007). Inhibitors of ubiquitin-activating enzyme (E1), a new class of potential cancer therapeutics. Cancer Res.

[10] Murai N, Shimizu A, Murakami Y, Matsufuji S (2009). Subcellular localization and phosphorylation of antizyme 2. J Cell Biochem.

[11] Murai N, Murakami Y, Tajima A, Matsufuji S (2018). Novel ubiquitin-independent nucleolar c-Myc degradation pathway mediated by antizyme 2. Sci Rep.

[12] Qin YR, Qiao JJ, Chan TH, Zhu YH, Li FF, Liu H (2014). Adenosine-to-inosine RNA editing mediated by ADARs in esophageal squamous cell carcinoma. Cancer Res.

[13] Okugawa Y, Toiyama Y, Shigeyasu K, Yamamoto A, Shigemori T, Yin C (2018). Enhanced AZIN1 RNA editing and overexpression of its regulatory enzyme ADAR1 are important prognostic biomarkers in gastric cancer. J Transl Med.

[14] Jain RK (2014). Antiangiogenesis strategies revisited: from starving tumors to alleviating hypoxia. Cancer Cell.

[15] Skobe M, Hawighorst T, Jackson DG, Prevo R, Janes L, Velasco P (2001). Induction of tumor lymphangiogenesis by VEGF-C promotes breast cancer metastasis. Nat Med.

[16] Waugh DJJ, Wilson C (2008). The interleukin-8 pathway in cancer. Clin Cancer Res Off J Am Assoc Cancer Res.

[17] Brat DJ, Bellail AC, Van Meir EG (2005). The role of interleukin-8 and its receptors in gliomagenesis and tumoral angiogenesis. Neuro Oncol.

[18] Moriconi A, Cesta MC, Cervellera MN, Aramini A, Coniglio S, Colagioia S (2007). Design of noncompetitive interleukin-8 inhibitors acting on CXCR1 and CXCR2. J Med Chem.

[19] Li A, Dubey S, Varney ML, Dave BJ, Singh RK (2003). IL-8 directly enhanced endothelial cell survival, proliferation, and matrix metalloproteinases production and regulated angiogenesis. J Immunol.

[20] Dwyer J, Hebda JK, Le Guelte A, Galan-Moya E-M, Smith SS, Azzi S (2012). Glioblastoma cell-secreted interleukin-8 induces brain endothelial cell permeability via CXCR2. PloS One.

[21] Schraufstatter IU, Trieu K, Zhao M, Rose DM, Terkeltaub RA, Burger M (2003). IL-8-mediated cell migration in endothelial cells depends on cathepsin B activity and transactivation of the epidermal growth factor receptor. J Immunol.

[22] Huang S, Mills L, Mian B, Tellez C, McCarty M, Yang XD (2002). Fully humanized neutralizing antibodies to interleukin-8 (ABX-IL8) inhibit angiogenesis, tumor growth, and metastasis of human melanoma. Am J Pathol.

[23] Shi Q, Abbruzzese JL, Huang S, Fidler IJ, Xiong Q, Xie K (1999). Constitutive and inducible interleukin 8 expression by hypoxia and acidosis renders human pancreatic cancer cells more tumorigenic and metastatic. Clin Cancer Res Off J Am Assoc Cancer Res.

[24] Karashima T, Sweeney P, Kamat A, Huang S, Kim SJ, Bar-Eli M (2003). Nuclear factor-kappaB mediates angiogenesis and metastasis of human bladder cancer through the regulation of interleukin-8. Clin Cancer Res Off J Am Assoc Cancer Res.

[25] Fousek K, Horn LA, Palena C. Interleukin-8: a chemokine at the intersection of cancer plasticity, angiogenesis, and immune suppression. Pharmacol Ther. 2020;219:107692. 10.1016/j.pharmthera.2020.107692 PMC8344087 32980444

[26] Lee YS, Choi I, Ning Y, Kim NY, Khatchadourian V, Yang D (2012). Interleukin-8 and its receptor CXCR2 in the tumour microenvironment promote colon cancer growth, progression and metastasis. Br J Cancer.

[27] Cao Z, Ding BS, Guo P, Lee SB, Butler JM, Casey SC (2014). Angiocrine factors deployed by tumor vascular niche induce B cell lymphoma invasiveness and chemoresistance. Cancer Cell.

[28] Li X, Coffino P (1994). Distinct domains of antizyme required for binding and proteolysis of ornithine decarboxylase. Mol Cell Biol.

[29] Chen H, MacDonald A, Coffino P (2002). Structural elements of antizymes 1 and 2 are required for proteasomal degradation of ornithine decarboxylase. J Biol Chem.

[30] Lopez-Contreras AJ, Sanchez-Laorden BL, Ramos-Molina B, de la Morena ME, Cremades A, Penafiel R (2009). Subcellular localization of antizyme inhibitor 2 in mammalian cells: Influence of intrinsic sequences and interaction with antizymes. J Cell Biochem.

[31] Mangold U, Leberer E (2005). Regulation of all members of the antizyme family by antizyme inhibitor. Biochem J.

[32] Madden SK, de Araujo AD, Gerhardt M, Fairlie DP, Mason JM (2021). Taking the Myc out of cancer: toward therapeutic strategies to directly inhibit c-Myc. Mol Cancer.

[33] Crews LA, Jiang Q, Zipeto MA, Lazzari E, Court AC, Ali S (2015). An RNA editing fingerprint of cancer stem cell reprogramming. J Transl Med.

[34] Zudaire E, Gambardella L, Kurcz C, Vermeren S (2011). A computational tool for quantitative analysis of vascular networks. PLoS One.

[35] Wang S, Xiong Y, Zhao L, Gu K, Li Y, Zhao F, et al. UCSCXenaShiny: an R/CRAN package for interactive analysis of UCSC Xena data. Bioinformatics. 2021;38:527–9. 10.1093/bioinformatics/btab561 PMC8723150 34323947

[36] Yu G, Wang LG, Han Y, He QY (2012). clusterProfiler: an R package for comparing biological themes among gene clusters. OMICS..

[37] Tang Z, Kang B, Li C, Chen T, Zhang Z (2019). GEPIA2: an enhanced web server for large-scale expression profiling and interactive analysis. Nucleic Acids Res.

