# Supplementary figures and images for: A novel mechanism for A-to-I RNA-edited AZIN1 in promoting tumor angiogenesis in colorectal cancer
Source: Cell Death Dis. 2022 Apr 2;13(4):294. doi: 10.1038/s41419-022-04734-8 (PMC8975946; doi:10.1038/s41419-022-04734-8)

Figure 1B

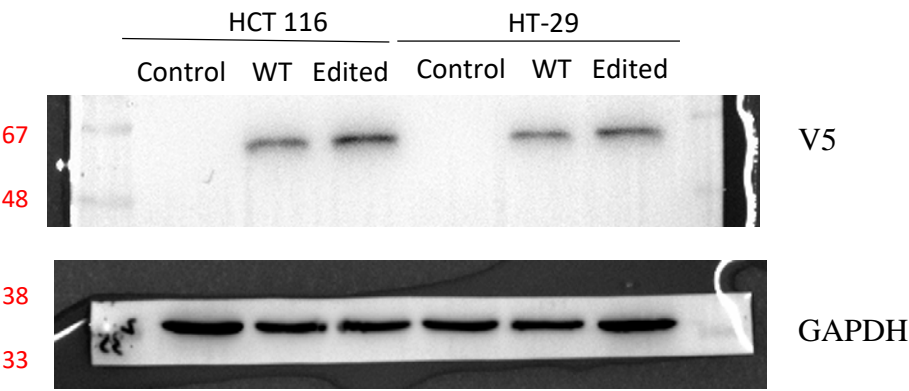

Fig. 4A

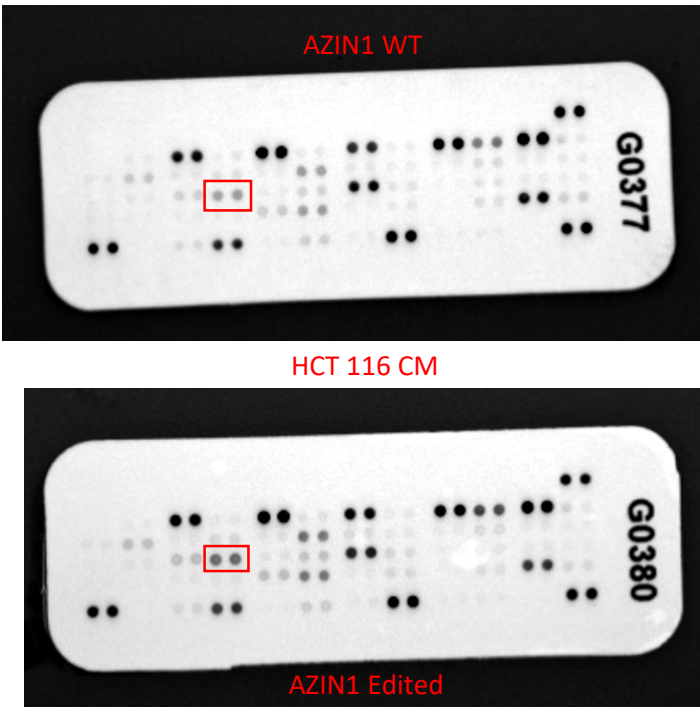

Fig. 4B

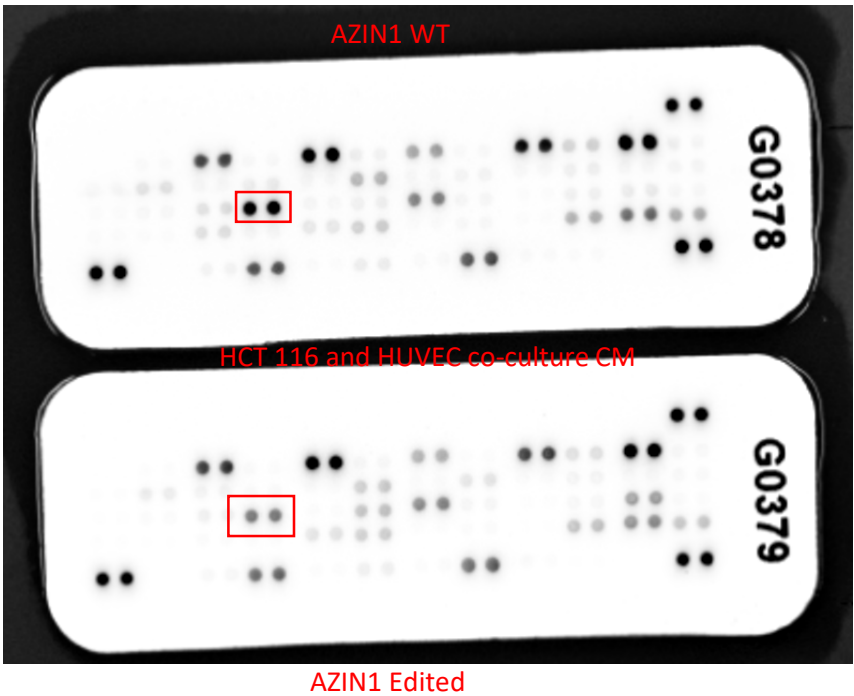

Fig. 7B

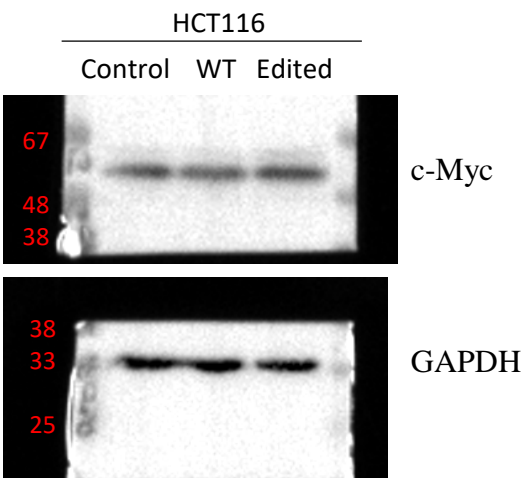

Fig. 7C

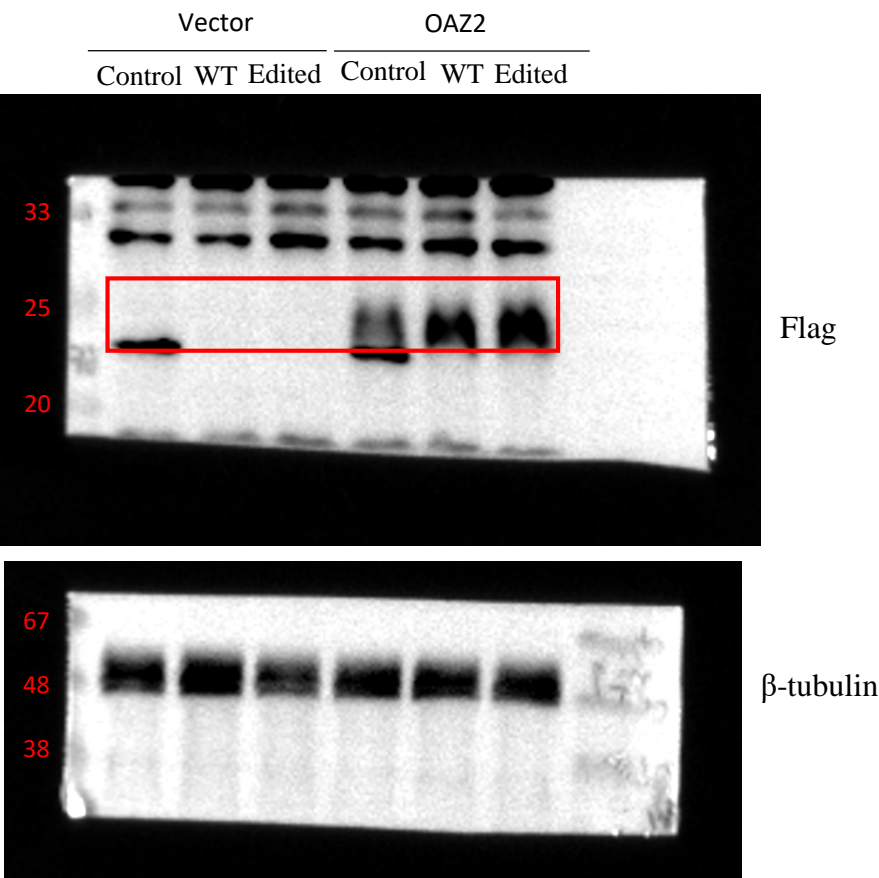

Fig. 7D

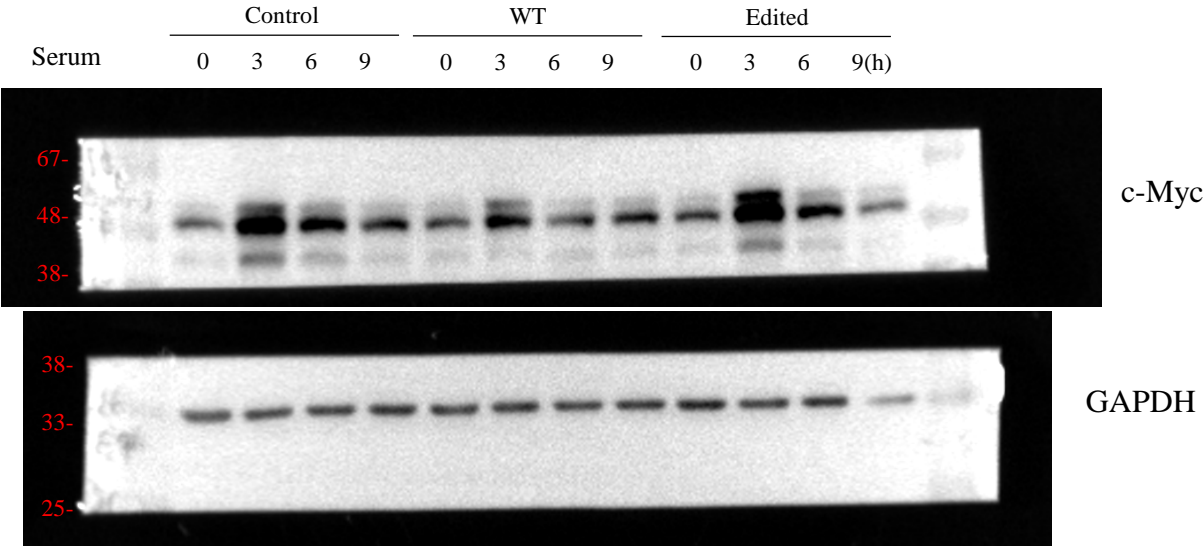

Fig. 7E

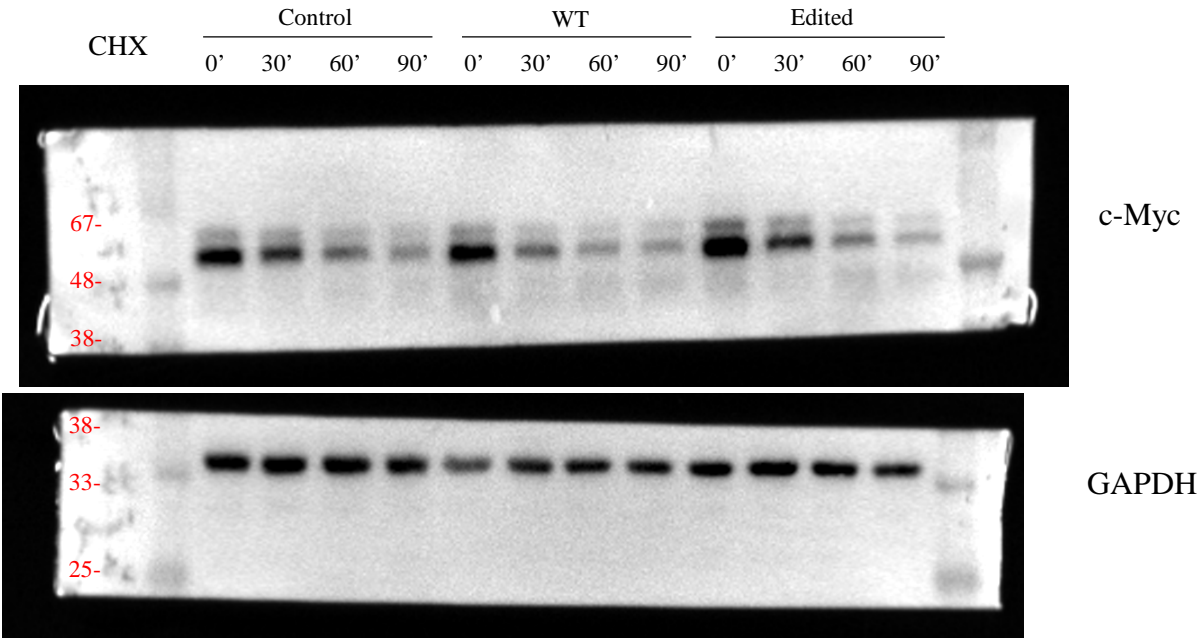

Fig. 7F

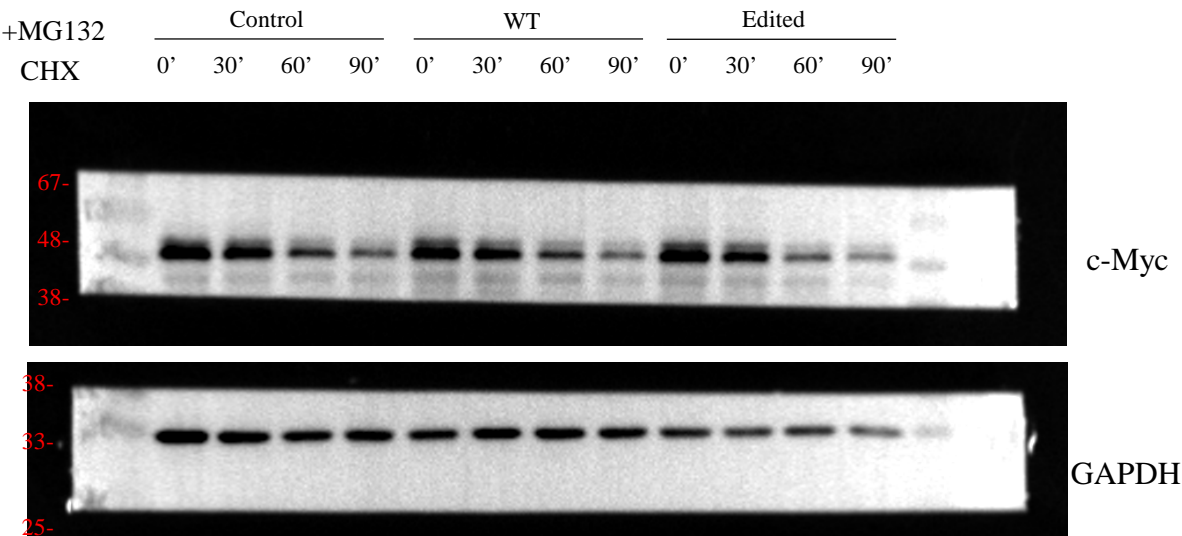

Fig. 7G

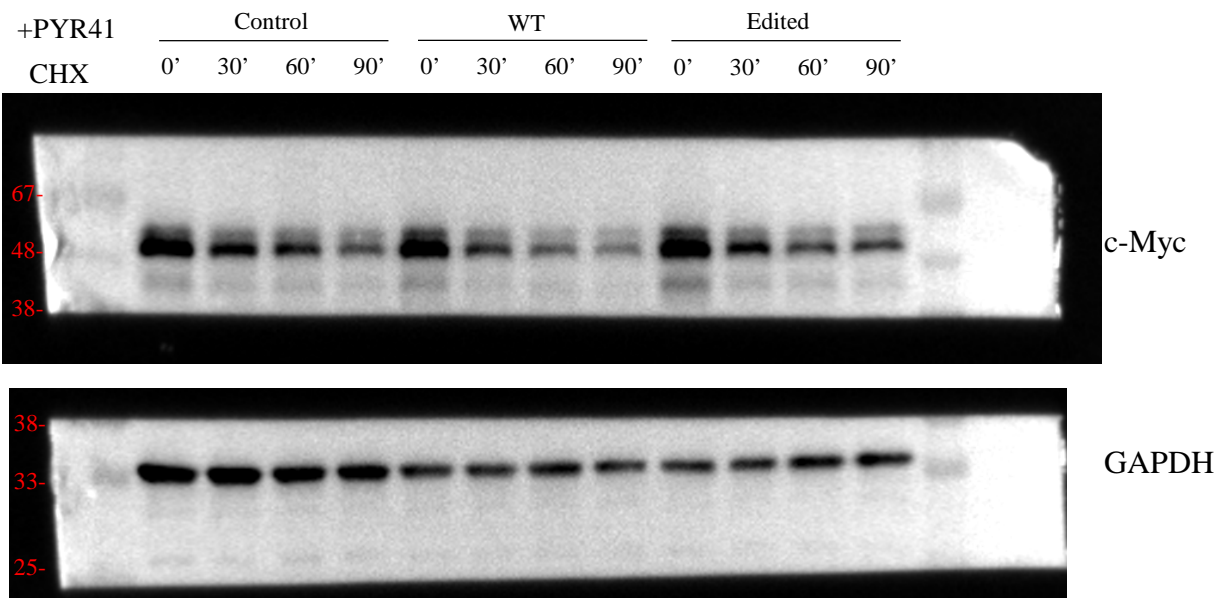

Fig. 7H

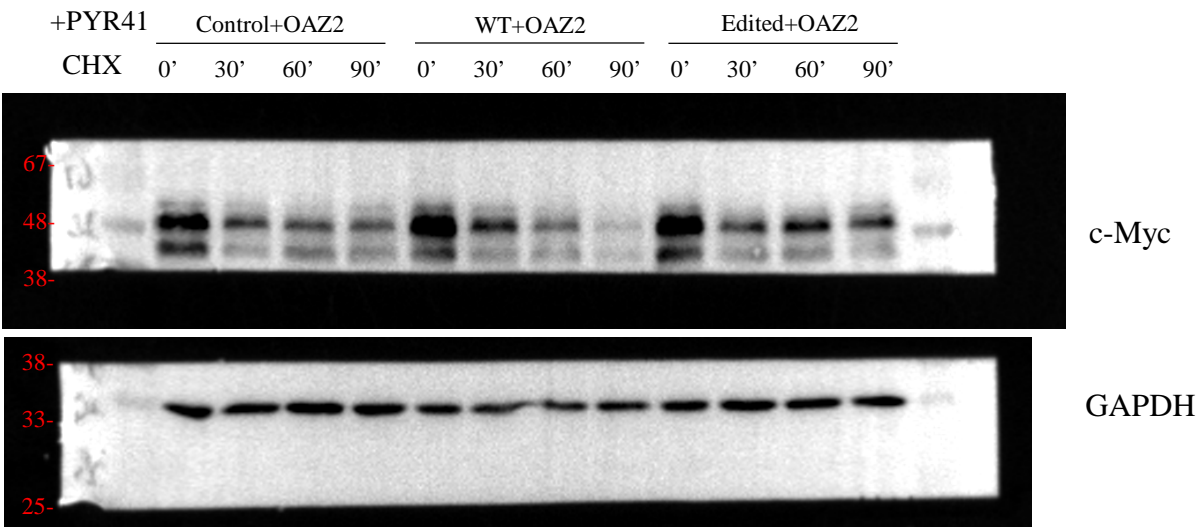

Supplement: Supplementary file 1 — Original western blots [file 41419_2022_4734_MOESM1_ESM.pdf]
